# Re-invent Yourself! How Demands for Innovativeness Reshape Epistemic Practices

**DOI:** 10.1007/s11024-021-09447-4

**Published:** 2021-06-08

**Authors:** Ruth I. Falkenberg

**Affiliations:** 1 Department of Science and Technology Studies, University of Vienna, Vienna, Austria; 2 Research Platform Responsible Research and Innovation in Academic Practice, University of Vienna, Vienna, Austria

**Keywords:** Research evaluation, Funding, Epistemic practices, Innovativeness, Competition, Research policy

## Abstract

In the current research landscape, there are increasing demands for research to be innovative and cutting-edge. At the same time, concerns are voiced that as a consequence of neoliberal regimes of research governance, innovative research becomes impeded. In this paper, I suggest that to gain a better understanding of these dynamics, it is indispensable to scrutinise current demands for innovativeness as a distinct way of ascribing worth to research. Drawing on interviews and focus groups produced in a close collaboration with three research groups from the crop and soil sciences, I develop the notion of a project-innovation regime of valuation that can be traced in the sphere of research. In this evaluative framework, it is considered valuable to constantly re-invent oneself and take ‘first steps’ instead of ‘just’ following up on previous findings. Subsequently, I describe how these demands for innovativeness relate to and often clash with other regimes of valuation that matter for researchers’ practices. I show that valuations of innovativeness are in many ways bound to those of productivity and competitiveness, but that these two regimes are nevertheless sometimes in tension with each other, creating a complicated double bind for researchers. Moreover, I highlight that also the project-innovation regime as such is not always in line with what researchers considered as a valuable progress of knowledge, especially because it entails a de-valuation of certain kinds of long-term epistemic agendas. I show that prevailing pushes for innovativeness seem to be based on a rather short-sighted temporal imaginary of scientific progress that is hardly grounded in the complex realities of research practices, and that they can reshape epistemic practices in potentially problematic ways.

## Introduction

In the current research landscape there is, along with the discourse on “excellence” and “frontier research” (see e.g. Flink and Peter 2018), a growing demand for research to be innovative, pioneering, or cutting-edge. While innovativeness and originality are central criteria in the peer review of publications (Siler and Strang 2017), and even associated with the moral qualities of a researcher herself (Guetzkow et al. 2016; Lamont 2010), positive valuations of *being innovative* are moreover institutionally embedded in the funding landscape through an increase in funding schemes, such as the European Research Council (ERC), that are explicitly oriented towards supporting “unconventional, innovative approaches and scientific inventions” (ERC 2019: 8) and research that is “ground-breaking” or “high-risk” (Heinze 2008; Laudel and Gläser 2014; Philipps and Weißenborn 2019). Yet, also national funding agencies increasingly demand research to be highly innovative and cutting-edge instead of just taking the “next logical step or the incremental further development of published data [which] is not considered to be innovative or original”.^1^

At the same time, concerns are being voiced that research may develop in the exact opposite direction. Since some years, scholars investigating the consequences of neoliberal governance regimes in the sphere of research and higher education (Lave, Mirowski, and Randalls 2010; Shore 2008) have noted that the metrification and growing competitiveness of academic science (Burrows 2012), as well as the shift towards project funding schemes, hamper scientific innovation (e.g. Franssen et al. 2018; Müller and de Rijcke 2017; Whitley, Gläser, and Laudel 2018). It has, for example, been described that the projectification of research with its particular temporal and organisational constraints dis-incentivizes researchers to deviate from organisational and epistemic standards (Franssen et al. 2018; Laudel 2006; Laudel and Gläser 2014) and that more generally, such changes in the governance of science leave researchers with less “protected space”, in terms of both time and resources, in which research is “shielded from interference” (Whitley, Gläser, and Laudel 2018: 113) and scientific innovation can develop. Furthermore, scholars have suggested that as project frameworks and competitive performance metrics create structural ties between epistemic uncertainties and personal risks (Sigl 2016), researchers are deterred from taking daring epistemic decisions where publishable output might not be guaranteed within a given timespan—thereby impeding “risky intellectual breakthroughs” (Fochler, Felt, and Müller 2016: 198; Müller and de Rijcke 2017). In this body of work, “innovative research” or “scientific innovation” are thus often evoked as desirable goals, but also as the red flag that is waved to highlight the problematic consequences of the dominant evaluative logics of the current research landscape and its fast-paced temporalities. Yet, it seems that in the existing literature *being innovative* has not been examined as a separate normative framework that researchers increasingly need to do justice to, and that they have to negotiate in relation to other evaluative criteria of research.

In this paper I thus start from the assumption that demands for research to be innovative—as an essential aspect of current regimes of research governance that has not received much explicit attention so far—need to be better understood. I suggest that it is, first, important to unpack innovativeness as a distinct way of ascribing worth to research, rather than only as something that is impeded in the current research landscape, or that is necessarily to be fostered. In order to better understand pushes for innovativeness, I draw on the lens of the “project order of worth” (Boltanski and Chiapello 2005; 2017), in which innovation, adaptability, and experimentation are appreciated. Based thereupon, I develop in dialogue with my empirical material the notion of a project-innovation regime that can be traced in the sphere of research—my empirical entry point being a close collaboration with three research groups from the crop and soil sciences.

Subsequently, I ask how the valuations embedded in the project-innovation regime relate to other forms of ascribing worth that were important to the researchers I talked to: First, I illustrate that innovativeness is often required for succeeding within the logic of *academic competitiveness and productivity*, which has frequently been described as the dominant regime of valuation in the current academic landscape (Burrows 2012; Fochler 2016; Fochler, Felt, and Müller 2016), but how these valuations are nevertheless sometimes in tension with demands for innovativeness, creating a double bind which is hard to navigate for researchers. Subsequently, I explore how demands for innovativeness relate to the seemingly most fundamental form of ascribing worth to science, namely the *progress of knowledge*. I argue that, contrary to prevailing positive connotations of innovativeness, current demands for being innovative are often in conflict with what researchers consider valuable for the progress of knowledge and for contributing to practical problems, and I illustrate some of the problematic epistemic consequences that arise from these dynamics. Furthermore, I show that prevailing pushes for innovativeness seem to be based on a rather short-sighted temporal imaginary of scientific progress that is hardly grounded in the complex realities of research practices. Last, I illustrate some of the strategies researchers use to navigate these conflicting evaluative logics in which they find themselves. These strategies partially allow scientists to conduct the kinds of research they actually find valuable, whereas sometimes, such creative workarounds are simply not possible.

Overall, the attempt of this paper to critically scrutinise demands for innovativeness in the current research landscape and their consequences for research practices may contribute to discussions about which kinds of research are and should be furthered through mechanisms of research governance—a discussion that is increasingly seen as indispensable by members of the academic community themselves (see e.g. Alberts et al. 2014; Curry 2018; Pagano 2017), and that seems all the more pressing in the light of growing socio-ecological crises.

## Valuing and Evaluating Research

Every research process can be understood as an act that involves assembling, aligning, and displacing different sets of values—when, for example, taking decisions about which questions to look at, which methods to choose, or how to set up an experiment (Dussauge, Helgesson, and Lee 2015; Helgesson, Lee, and Lindén 2016). In their work, researchers thus constantly relate to different evaluative principles such as the relevance of their research to a certain practical problem, the publishable output it generates, or the degree of cooperation they want to integrate in their practices. Since such different valuations do often not neatly align, researchers may order dissonant principles in certain ways or create compromises between different value orders (Dussauge et al. 2015; Rushforth et al. 2019)—which can result in more “heterarchical” or more hierarchical, i.e. polarised, orderings (Stark 2009).

Scholarship studying (e)valuation practices in contemporary academia has pointed out that along with the growing importance of various metrics and competitive performance measures (Felt 2009; Hazelkorn 2011; Lave, Mirowski, and Randalls 2010; Shore 2008), evaluative principles in academic science are becoming narrower and dominated by easily quantifiable criteria (Burrows 2012; Fochler, Felt, and Müller 2016). As various authors have described, the competitive dynamics of the academic sphere take increasingly market-like forms, and it appears that capitalist cultural logics, detached from direct economic relations, are becoming more and more pervasive in the scientific landscape (Fochler 2016; Hackett 2014; Kleinman 2010; Mirowski 2011). Therefore, previous studies have conceptualised principles of competitiveness and productivity in the academic sphere not only as a dominant *regime of valuation* but also as one of *accumulation*, inciting researchers to make worth durable in the form of publications or employability that can be used as assets on the competitive academic market (Fochler 2016; Fochler, Felt, and Müller 2016; Muniesa et al. 2017; Pinel 2020). This dominant regime of valuation/accumulation is structured by the fast-paced temporalities of the projectified academic landscape where researchers are typically employed on short-term contracts, thus requiring them to ensure the accumulation of academic capital within these given timeframes.

Studies that have adopted actor-centred perspectives to understand how researchers navigate this hierarchical value landscape (see e.g. Felt 2009; Müller 2014; Müller and Kaltenbrunner 2019; Sigl 2016) have highlighted that as scientists are socialised into the competitive academic environment, the evaluative principles they draw upon become increasingly limited and oriented towards a focus on productivity in terms of publishing in highly ranked journals, and obtaining grant money and citations (Felt 2009; Fochler, Felt, and Müller 2016). At the same time, other concerns related to, for example, societal responsibility or care and cooperation with colleagues are often being overridden (Åm 2019; Sigl, Felt, and Fochler 2020). Moreover, as noted before, also researchers’ epistemic decisions are becoming structured by currently dominant evaluative logics and their particular temporalities, and as a consequence, the pursuit of innovative research is said to be impeded (Fochler 2016; Franssen et al. 2018; Müller and de Rijcke 2017).

However, and this is the starting point of this paper, innovativeness in itself has so far not been considered as a distinct way of ascribing worth to research that plays an increasingly important role in the current scientific landscape, and that researchers need to align with the dominant logic of competitiveness and productivity, but also with other regimes of valuation.

## The Project-Innovation Regime as a Particular Form of Ascribing Worth

In this paper, I suggest that it is fruitful to not only treat innovativeness as a dependent variable, i.e. as something that is impaired by current academic valuation logics, or as something that should be necessarily fostered, but as an evaluative regime in its own right. In order to conceptualise innovativeness as a distinct way of ascribing worth to research, I borrow from Luc Boltanski and Eve Chiapello (2005, 2017) who have identified the *project order of worth* as a specific justificatory regime, being characteristic of a *new spirit of capitalism* that has arisen since the 1980s.^2^ As put by Tamar Sharon (2018: 7), “mobilizations of the project repertoire will be at stake whenever notions like experimentation, innovation, thinking outside of the box and shaking things up are promoted as valuable”. Within this order of worth, a “great one” is an innovative visionary and leader, being able to completely devote her enthusiasm to a certain project, but also capable of reversing and shifting her creativity and excitement and to commit to an entirely new project (Bröckling 2007). As such, “[l]ife is conceived as a series of projects, all the more valuable when different from one another” (Boltanski and Chiapello 2005: 169). In contrast, a “state of smallness” is marked by rigidity, stability, and an unwillingness to adapt and move on to new projects.

In this paper, I explore in how far valuations akin to the project order as described by Boltanski and Chiapello can be traced in researchers’ discussions on demands for innovativeness in the current research landscape. However, I do not treat this framework as a blueprint to be applied to the sphere of research. Rather, I trace comparable tendencies in researchers’ narratives without attempting a full-fledged analysis of how the project order can be found in the academic world.^3^ Therefore, I developed, in dialogue with my empirical material, the notion of a *project-innovation regime,* to denote tendencies comparable to those identified by Boltanski and Chiapello in their analysis of management literature, but to foreground the particular focus on innovativeness that I adopt in this paper.

My conceptual approach is thus situated somewhere between studies that have applied pre-defined orders of worth (Boltanski and Thevenot 2006) as a framework to be traced in different societal spheres (e.g. Sharon 2018; Thévenot, Moody, and Lafaye 2000), and approaches that have empirically reconstructed regimes of valuation (Fochler, Felt, and Müller 2016) or registers of valuing (Heuts and Mol 2013) in a bottom-up manner (Dussauge, Helgesson, and Lee 2015; Helgesson, Lee, and Lindén 2016; Stark 2009). While I applied the lens of the project order, I also traced how it concretely played out in my empirical material, as what I call *the project-innovation regime*. Moreover, I investigated how valuations of innovativeness are related to, potentially in tension with, and being aligned with different value regimes that I identified to be of importance to the researchers I talked to in a bottom-up manner in the process of data analysis. For my analysis, I employ the notion of *regimes of valuation*, rather than *repertoires* or *registers*, since this concept most strongly highlights the discursive, material, and institutional embedding that concrete evaluative principles draw upon (see Fochler et al. 2016).

## Case, Approach, and Methods

The argument of this paper draws on material produced in the scope of the project *Valuing, Being, Knowing. Understanding the entanglements of valuation practices and subjectification processes in life science research*. The project is based on a close collaboration with three research groups working in the fields of plant breeding, soil/plant chemistry, and soil microbial ecology, and it employs a range of methods including semi-structured qualitative interviews, observations, focus groups, and document analysis. A participatory approach, the ongoing collaboration with the groups over a time period of three years, and the triangulation of methods aim to enable an in-depth understanding of how researchers take decisions in their practices, and of how valuations of scientific research are intertwined with the epistemic directions researchers take.

Being embedded in this framework, the argument presented here is equally based on a range of different materials. The basis of this material is given by 24 biographical interviews and 14 follow-up interviews with researchers across various career stages as well as observations and more informal conversations during field- and laboratory visits, group meetings, and at a conference in February 2020. Interviews and observations were conducted by myself, Maximilian Fochler, and Lisa Sigl between October 2019 and May 2020. These observations and the qualitative, semi-structured interviews were designed (1) to acquire an in-depth understanding of the researchers’ epistemic agendas, (2) of how their careers and epistemic agendas have developed over time, and (3) to map the various aspects that matter to them in taking decisions in their practices. During the analysis of these materials, I identified the issue of innovativeness to be an important concern for the participating researchers and I decided to further inquire into this topic through focus groups.

Three focus groups, respectively with one of the participating research groups, were conducted in June, July, and August 2020 by myself and Maximilian Fochler.^4^ The discussions were designed to, first, allow the researchers to more openly brainstorm different ways in which research could be innovative. Second, the participating researchers were asked to discuss which kinds of innovativeness they saw as being valued in the contexts of funding and publication, and to subsequently compare between these different contexts. Moreover, researchers were invited to compare these valuations of innovativeness in the research landscape with what matters to themselves, especially with regards to research they consider as good or relevant. This was followed by a more modular round in which, from a range of stimuli, selected ones were fed to the group to foster a more focused discussion on issues that the facilitators considered most relevant based on the preceding discussion.

The data were analysed through grounded theory-informed open coding (Charmaz 2006). In an iterative process going back and forth between data and literature, I identified resemblances between forms of innovativeness that researchers saw as demanded in the current research landscape and the project order of worth as described by Boltanski and Chiapello (2005; 2017). I explored these similarities further through theoretical coding (Rivas 2018) and developed the notion of the *project-innovation regime*. The other regimes of valuation I describe below—i.e. *academic competitiveness and productivity*, and the *progress of knowledge*—were largely reconstructed through bottom-up coding of the empirical material, though I also analysed valuations of competitiveness and productivity in comparison to previous literature.

Overall, because my approach is based on a rather small sample, my findings should not be uncritically extrapolated to other scientific disciplines, especially given that understandings of innovativeness and the ways in which researchers perceive and negotiate different sets of values differ between scientific disciplines (Barlösius 2018; Dussauge, Helgesson, and Lee 2015; Guetzkow, Lamont, and Mallard 2016; Kjellberg and Mallard 2013). Yet, an important advantage of the approach presented here is that the long-term collaboration with research groups enables a detailed understanding of their work, thereby allowing to link general valuations of innovativeness to concrete consequences for epistemic practices. Furthermore, the specificities of the three groups in terms of different institutional contextualisation, different funding and staff situation, different epistemic conditions (e.g. different temporalities of the primary epistemic objects), as well as an embedding in more ‘basic’ vs. more ‘applied’ research contexts,^5^ also allow to highlight how broader evaluative logics come to matter in different sub-disciplines of the life sciences.

## Empirical Findings

In the following, I first describe how I saw the project-innovation regime expressed in researchers’ accounts. Subsequently, I illustrate how researchers talked about these valuations of innovativeness in relation to valuations of academic competitiveness and productivity. I highlight that although the project-innovation regime and that of productivity/competitiveness are in many ways deeply intertwined, they nevertheless pose sometimes conflicting demands to researchers. Furthermore, I describe how valuations of innovativeness are related to, and sometimes in tension with another evaluative regime that was very important to my interlocutors, namely the progress of knowledge, and I relate this to conflictual temporal imaginations embedded in these two regimes. Last, I illustrate some of the strategies researchers use to negotiate these partially conflicting valuations in their work.

## The Project-Innovation Regime in Researchers’ Accounts

The three participating research groups are all well-situated within their epistemic communities and they are actively engaged in exploring and developing innovative approaches in their fields, two of the group leaders also being ERC grantees. At the same time, they perceived *being innovative* as a strong demand in the current scientific system. In researchers’ discussions, demands for innovativeness were frequently talked about as (1) an appreciation of *taking first steps* rather than second ones and (2) the need to constantly *re-invent oneself*.

### Taking First Steps

When researchers discussed demands for being innovative in the current academic landscape, their narrations frequently pointed to a perceived need for *taking first steps* rather than second ones—valuations that they saw as being particularly often applied to publications. This came along in different shapes in the different research groups. Researchers working in the field of plant breeding described that while in their field, studies would frequently map genetic loci for certain plant traits, few of these were followed up on in validation studies. Reflecting on this issue and the reasons for it, one researcher noted that

validation studies are less attractive than the first step, so many papers are published and they propose something, but nobody follows up, validates, because you don’t get a good reputation for a validation study. You always get more reputation for a first-time publication and not for proving that it’s true, or showing that it’s possibly only half true. It’s not valued in our community, or in the general community. (Senior scientist/PI)

Researchers from the field of microbial ecology pointed to a comparable phenomenon in their field, noting that it seemed to be of less value to follow up on hypotheses which researchers established in previous publications. More concretely, this could, for example, mean that a certain function of a microorganism would be suggested based on genomic data, yet the validation of this hypothesis based on an analysis of the actual organism was deemed less important in the field and therefore most likely could not be published as well as the original suggestion. One Post-Doc described that, when nevertheless having done such a follow-up study and aiming to publish it, reviewers had commented that this was not novel enough. One of his colleagues then noted that:

these days it appears to be enough to just speculate an organism is doing something. Once it’s speculated in the literature, the race is won and we move on the the next. At times, it appears that the actual testing of working hypotheses is not per se necessary, that’s yesterday’s news. (Senior scientist/PI)

### Re-inventing Oneself

Furthermore, especially with regards to the evaluative contexts of funding and career advancement, researchers’ discussions often pointed to a need to *re-invent oneself*, indicating a demand for innovativeness that is closely related to the appreciation of *taking first steps*, yet different in its nuances and focus. This aspect centres more specifically around the person of the researcher herself, pointing to a perceived demand to constantly innovate the own work and, in a way, oneself—moving on to new topics, instead of becoming overly attached to a certain problem. Researchers, for example, noted that as a scientist, “you constantly need to challenge yourself. You cannot simply continue to work with what you have established and what is functioning well. This is not the way research works” (Senior scientist/PI).

This way of ascribing worth was often noticeable when researchers discussed what they saw as being valued by funding schemes such as the ERC. Targeting “independent researchers” who put forward “pioneering proposals addressing new and emerging fields of research” (ERC 2019: 8), instead of simply continuing an established line of work, such funding schemes indeed seem to institutionally embody the demand to re-invent oneself. Yet, researchers also perceived an increasing push into a similar direction from national funds, where it would equally become more difficult to ‘just’ continue an established trajectory.

Next to this embedding in the context of funding, researchers described more tacit career-related dimensions of the need to re-invent oneself. For example, researchers mentioned that it could be valuable for one’s career to make oneself independent from the former supervisor after the PhD or to head into a new area after the first Post-Doc position, because such changes would make oneself a more “well-rounded scientist”. Some researchers even framed it as a “philosophy” prevailing in certain scientific institutions that one should not stay at a certain place too long but always move on to new places and topics. As such, showing flexibility and independent spirit could be beneficial for acquiring new positions. As one participant described:

during the application it turned out in my favour, because they thought that if this person does it, the person must be, you know, motivated and kind of adventurous, and I also had already some changes of subject, of different things that I’ve done in Bachelors and so on, and this was how I got the position there as a PhD. (Post-Doc)

Overall, the project-innovation regime as reconstructed from my data is thus characterised by a constant striving for the radically new. This means, in publications, to offer first steps into novel and unexplored areas rather than following up on previous results, and, with regards to projects submitted for funding and career trajectories, to constantly re-invent oneself and move on towards new and innovative topics. Valuations of innovativeness hence seem to be applied to research *endeavours*, to *products,* i.e. publications, as well as to researchers themselves and their *careers*.^6^ Given the discursive and institutional embedding of these valuations, they can indeed be conceptualised as a *regime of valuation* that exerts normative power over researchers’ decision and actions.

## Aligning the Project-Innovation Regime with Other Forms of Worth

As became clear from researchers’ accounts, the valuations embedded in the project-innovation regime are often in an ambivalent or even conflictual relationship with other ways of ascribing worth to research. In the following, I discuss the project-innovation regime in relation to, first, valuations of competitiveness and productivity, and second, to valuations centring around the progress of knowledge.^7^

### Negotiating Innovativeness and Individual Competitiveness/Productivity

Previous studies have identified valuations of competitiveness and productivity as the dominant regime of valuation in the current academic sphere (Burrows 2012; Fochler, Felt, and Müller 2016), and such considerations were indeed highly present in researchers’ discussions. On the one hand, it seems that these valuations often align with those embedded in the project-innovation regime. Since researchers described that re-inventing oneself is appreciated when it comes to acquiring grant money and job positions, and first step publications instead of validation studies are publishable in more highly ranked journals, this indicates that these kinds of innovativeness help researchers, or are even required, to accumulate resources in terms of publications, grants, or employability and to succeed on the competitive academic market.

Yet, the relation between the project-innovation regime and the regime of competitiveness/productivity is more complex. First, previous productivity in terms of publications demonstrating expertise on a certain topic was usually seen as a requirement for having the very chance to do innovative work. For example, when applying for funding, the appreciation of being innovative and venturing into new areas is combined with the demand for demonstrating a considerable track record on a certain topic. This creates a double bind which is often hard to manoeuvre for researchers since, naturally, when aiming to explore a very novel topic, one may not have done much work on it before. One researcher nicely pinpointed this friction:

So in the grants that I’m putting forward, I’m proposing research avenues with aspects that I don’t have much experience in—but I have tried to align myself with external collaborators that can contribute their expertise to the research. As such, I thought it was a cool project backed with leading experts in this area and state-of-the-art methods, innovative and advancing the field. But then I get criticism back that’s saying: well, you have no expertise, you have no knowledge about this, so what makes you think we should give you funding for that? (Senior scientist/PI)

As this quote shows, previous resources are often indispensable for acquiring funding for innovative research endeavours. Yet, researchers discussed that it was at the same time required to hold a fine balance in this regard, since having done too much previous work on a topic could be perceived as “just the continuation of what you have established” and make a research endeavour appear less innovative.

A similar dynamic was described concerning the demonstration of preliminary data—which researchers usually saw as necessary to ensure that an innovative research project will be do-able and deliver the envisaged output, whereas showing too much preliminary data could undermine its innovativeness. Researchers however noted that limited resources in terms of time and staff would often make it difficult to produce such preliminary data alongside other projects. Furthermore, it was highlighted that innovativeness often goes along with “high risk”—when taking steps into the unexplored, the outcomes are naturally very uncertain. This then creates another double bind that is extremely difficult to navigate:

isn’t it a dilemma that […] you need something ground-breaking and innovative, which means you don’t know what will come out, but the funding agency also wants some security that the project will work, so they need already some preliminary results. But if it’s like too secure, it cannot be innovative because then you already have enough evidence that this will probably work. Isn’t it contradictory? (Post-Doc)

As indicated by this quote, doing very innovative research and courageously venturing into novel areas is sometimes not only in tension with the need to demonstrate *previous* academic capital and experience, but potentially also with what is required to *remain* academically competitive, namely conducting ‘safe’ research that is publishable within a given timeframe.

This friction was described in the context of funding, but also with regards to moving into new fields in one’s career more generally. While, as noted before, re-inventing oneself and changing the field one is working in was, within the project-innovation regime, seen as a valuable step that was often demanded on the academic market, researchers highlighted that this would entail considerable difficulties when it comes to ensuring ongoing future productivity. As one participant put it, venturing “into something new […] throws you back to a state of origin”, therefore requiring a lot of work and nearly inevitably entailing a gap in publications. A gap in one’s publication record can, in turn, be problematic for receiving further funding, when applying for new positions, or when aiming for a habilitation. As such, moving into a new field “does certainly, hands down, come at a cost—and the cost is not showing productivity compared to competitors, who don’t have that gap because they stayed in their field. But these are the people you’re competing with for permanent positions” (Senior scientist/PI).

Overall, prevailing demands for innovativeness thus stand in a very ambivalent relationship with valuations of academic productivity and competitiveness. On the one hand, these two regimes of valuation are tightly bound to each other, yet researchers simultaneously experience fundamental frictions between them, which can indeed sometimes hinder the pursuit of innovative research. An important argument of this paper, however, is that not only the constriction of innovativeness by logics of productivity and competitiveness is to be considered problematic. Rather, as became clear from researchers’ discussions, it is equally indispensable to critically scrutinise demands for innovativeness themselves with regards to their consequences for research practices and for the *progress of knowledge*.

### Tensions Between Demands for Innovativeness and the Progress of Knowledge

The *progress of knowledge*^8^ is probably the most fundamental way in which worth can be ascribed to science, and such valuations played a central role in participants’ narrations. Even though not being discussed as such in previous literature, valuations related to the *progress of knowledge* can be understood as another regime of valuation, given their central embedding in scientific institutions, imaginations, and discourses around research. In the following I illustrate that, even though it may seem intuitive that for the progress of knowledge it is valuable and important for research to be innovative, the valuations of the project-innovation regime do not always coincide with what researchers considered necessary for advancing knowledge or for becoming relevant to practical problems.

On the one hand, researchers described both re-inventing oneself and taking first steps into new fields as valuable in certain situations or for particular questions. For example, it was noted that “there is a benefit for a research field if people with different backgrounds join this field. They bring new ideas and with this also innovation is going to happen” (Senior scientist/PI). Different researchers described that, even though it would require a certain courage to move to new fields or topics, this could be exciting and inspiring and the new perspectives brought in by this could be essential for gaining a more fundamental understanding of a certain problem.

On the other hand, researchers noted that if the philosophy of having to move on towards new topics and re-inventing oneself would become too dogmatic, this could become problematic for advancing knowledge. For example, it could mean that “projects are just disrupted, just because people need to move on, and they just leave because that’s the philosophy, and sometimes expertise is lost with these people because they don’t manage to transfer it” (Post-Doc).

Whereas this quote points to the problematic consequences of the need to re-invent oneself and move on to new places and topics in terms of job positions, researchers described similar tensions arising when demands for innovativeness and moving on to radically new topics would become too imperative in the context of funding:

That is such a pity, we have such nice data now, and most of all a good understanding of the system. We have done extremely important work, everything you need to continue in a really productive manner. And now I am at the point, if I do not soon get further funding, then I might have to phase this out. Then all the work that we have done in all those years will not fully pay off. We have established databases, through sequencing we know which microorganisms are to be found there, what they might be able to do and we have postulated how they survive and which strategies they use. And I would like to further test all this now, but therefore I need money again. And if I do not get that money, then the whole thing can only continue on a very low flame, or will entirely phase out. And that would be really sad, because you cannot always build something like that from scratch with every project, you need to be able to build on that. (Senior scientist/PI)

Here, the criticism of needing to re-invent oneself rather seamlessly blends into that of considering first steps as more worthy as second steps and follow-up work. Researchers described that while it could surely be valuable to take first steps into entirely novel directions, it would nevertheless impede the progress of knowledge if continuous work and follow-up studies were not done because such research was not considered innovative anymore. Indeed, when being confronted with a quote from the guidelines of an Austrian funding agency stating that “the next logical step or the incremental further development of data is not considered to be innovative or original”, discussions became very agitated and researchers across different groups and career stages expressed considerable concern and frustration:

So I—this quote upsets me as a scientist and I’ll tell you why: what we do in our research group is a nice balance between looking at genomic potential, […] and then we come up with working hypotheses and how a system could be working. And the next logical step or the increment to further develop that data would be to then actually test it with strains or microorganisms in the lab, because just because you find genetic potential that does not mean that it manifests physiologically in the environment. (Senior scientist/PI)

In line with this quote, researchers expressed fundamental concerns that if funding agencies’ demands such as the one quoted above would be taken at face value, science could end up in a vicious circle where researchers would try to “push” their results in order to suggest innovative and exciting hypotheses in publications, which however remain untested since subsequent studies need to move on to explore more fundamentally novel aspects. Yet, as different researchers highlighted, such postulated hypotheses may often, although not further validated, still become established assumptions that future research works with—a dynamic that participants were highly worried about.

Importantly, while researchers saw demands for being innovative in the sense of taking first steps and re-inventing oneself as disconcerting with regards to the progress of knowledge as such, scientists from the field of plant breeding highlighted that these dynamics would moreover become problematic when it comes to the further evaluation of scientific results for their practical relevance. As noted before, researchers from this discipline described that validation studies to further evaluate previously mapped genetic loci for certain plant traits were often not done because they were not considered innovative enough. Therefore, as one PI highlighted, thousands of studies mapping certain genetic loci for the first time were “residing in bookshelves”, but very little of these would end up in cultivars on the field—a dynamic with which both researchers and people from the practical breeding community were very dissatisfied.

## Conflicting (Temporal) Imaginaries of Scientific Progress

What constantly surfaces in the description of these tensions is a fundamentally different imagination of scientific practices and their temporalities that seems to underlie the valuations of the project-innovation regime, on the one hand, and researchers’ own understanding of their work, on the other hand. Demands to constantly move on to entirely novel things seem to imply, as one participant put it, “this idea, this thought process of: you just need to be the first to suggest it, and boom—it’s done” (Senior scientist/PI). If researchers are required to do highly innovative work with every single paper or project (which in turn needs to result in publishable output), this seems to assume that innovative research does not need much preparatory work and that an innovative project can well be brought to a satisfactory end in a timeframe of three or five years. What is implicated here is thus a very short-lived temporal imagination of research practices, where scientific progress seems to be conceived as simply going “from milestone to milestone”.

This imaginary differs fundamentally from researchers’ own perspectives, who put forward much more long-term, complex, and distributed understandings of scientific processes and, indeed, progress. As researchers pointed out, instead of coming about in single big leaps that are achieved in the scope of individual research projects or papers, important scientific achievements are usually “incremental rather than game changers—sometimes we have a big jump, but most of the times you go rather small steps forward, and failures” (Senior scientist/PI). For the researchers, this in turn meant that an innovative research endeavour is not necessarily one that achieves such a big milestone, but that research which slowly and progressively builds a base of knowledge could be equally valuable and, indeed, innovative at a different scale. Some researchers moreover put forward a conception of their own role in the overall progress of knowledge that seems to be very different from the image of the brilliant scientist coming up with a breakthrough finding within the framework of a single project. As one researcher, notably an ERC grantee, put it, “I think that every breakthrough, brilliant insight builds on much much more […] and I don’t think that I, or my research will deliver such breakthrough insights, but I think that I can contribute to what leads there” (Senior scientist/PI).

Overall, demands for innovativeness in the sense of constantly moving on and re-inventing oneself are thus in tension with what researchers saw as valuable for the progress of knowledge not because researchers do not appreciate innovativeness, but because such demands entail a systematic *de-valuation* of second steps and hence of the pursuit of long-term epistemic agendas that seems to be hardly grounded in the actualities of scientific practices. One researcher illustratively captured this discrepancy: “We as scientists are essentially building this wall of knowledge, and so every research project is a separate brick in this wall of knowledge. So how this is going, are we simply just making a frame and not filling in the details of this wall?” (Senior scientist/PI).

## Doing Research Within Conflicting Evaluative Logics

An inevitable question that arises from this analysis is how researchers actually deal with these conflicting valuations. While scientists are in a way subjected to dominant evaluative regimes in the academic sphere, they are also active agents in navigating these value frameworks, making compromises between different regimes of valuation (Fochler, Felt, and Müller 2016; Rushforth, Franssen, and de Rijcke 2019), and moulding their epistemic living spaces (Felt 2009). As such, they may try to orient their work along with evaluative principles that matter for themselves, such as the kind of scientific progress they consider important for gaining an in-depth understanding of a certain research problem, for achieving practical relevance, but also for staying motivated about their research. It is thus important to ask in how far researchers adapt their work to the valuations of the project-innovation regime or whether they also find creative workarounds to align this regime of valuation with other ways of ascribing worth to research.

First, researchers often described to *frame* certain elements of their overall research as being particularly innovative in order to receive funding for a research endeavour that would not be that innovative in the sense of completely re-inventing oneself or taking a first step into a novel area, but that they would consider a valuable continuation of previous work. As one researcher put it, when reflecting on the demand for not just taking “the next logical step”:

you have to make it look like it’s not like this, but in 99 percent of the cases your projects are like this, because you are at a certain step and that’s research, yeah? You try to answer one question but you open ten new questions, and that’s the next incremental steps, […] you always go from where you are; and I would say 99.9 percent of the projects are like: I do the next step now, instead of: forget what was before, I start something new, nobody thought about this before. (Senior scientist/PI)

As noted above, especially in project applications, this strategy of selling the own work as innovative of course needs to be constantly balanced with simultaneously showing the right amount of preliminary results in order to rhetorically demonstrate do-ability and hence do justice to demands for productivity and academic competitiveness (see e.g. Serrano Velarde 2019).

Besides this, an approach to actually increase the innovativeness of a research endeavour that researchers described was the addition of a high risk work package on top of the more safe work that would actually have been planned (see also Franssen et al. 2018). Similarly, a strategy for making a case for the innovativeness of a project could be the application of very innovative research technologies, or framing it in relation to certain hot topics. Importantly, these approaches allow researchers to combine demands for innovativeness and those for continuous productivity and competitiveness within a single research project, because they do not change the core of the project in a riskier direction, but simply add certain innovative elements to it.

While these strategies that aim at enhancing the innovativeness of a certain research endeavour in order to receive funding for it are playing within the dominant logics of the research landscape, another strategy that side-steps these logics is to pursue different lines of research next to funded work. This can, as described previously, allow to do research that is too innovative and risky to be reconciled with demands for guaranteeing output (Sigl 2016). Yet, such workarounds are equally crucial for conducting research that is too incremental to be considered worthy of funding or publication. This strategy was, for example, described as very important by researchers in the field of plant breeding, where relevant insights would usually require years and even decades of continuous work because of the long growth periods of plants on the field. As one scientist put it:

If I tell them at the [Austrian funding agency] that I will start with crossing and mapping experiments, then the reviewers will say—where is the novelty? Everybody can do this. I mean, the [Austrian funding agency] always says you have to be at the cutting-edge. (Senior scientist/PI)

Such rather incremental work using established methods and lines of thought would thus usually be done next to other kinds of projects. This strategy of creating a “portfolio” of different lines of work then allows researchers to do justice to multiple evaluative regimes simultaneously (Rushforth, Franssen, and de Rijcke 2019). However, whether such workarounds are possible depends on the situation of a certain research group and their reliance on different kinds of resources. While it was possible for the group of this particular PI from the field of plant breeding because they have a reasonable amount of permanent positions including a lab technician, such strategies were described as much more difficult by another PI who is mostly dependent on third-party project funding. As the latter PI described, if they would not get another grant for the continuation of a previous project where they had built extremely valuable resources, they would most likely not be able to continue this line of research. Overall, while researchers thus certainly have room for manoeuvring and acting strategically in order to do the kinds of research they find valuable, the possibilities for doing so are sometimes ultimately limited.

## Conclusions

The aim of this paper was to analyse currently omnipresent demands for innovativeness as a distinct evaluative regime, and to investigate its relation to other regimes of valuation that matter for researchers’ practices. To do so, I have drawn on the lens of the project order of worth which Boltanski and Chiapello (2005, 2017) have described as pervasive in contemporary western societies, and I have in dialogue with my empirical material developed the notion of a *project-innovation regime*. As I have shown, this regime of valuation plays a central role in the sphere of research, where it can be traced in a perceived need to offer first step publications instead of following up on previous findings, and to constantly re-invent oneself and move on to novel topics, particularly in the contexts of funding and of career advancement. As such, also scientific careers and processes of research seem to be “conceived as a series of projects, all the more valuable when different from one another” (Boltanski and Chiapello 2005: 169). This parallels broader societal “pro-innovation biases”, where breakthroughs and radical innovation are equally valued more than “imitation and the incremental” (Godin and Vinck 2017: 320).

Furthermore, I have illustrated that while innovativeness is frequently required for succeeding on the competitive academic market, researchers’ possibilities for doing innovative work are the same time bounded by dominant valuations of competitiveness and demands for previous as well as continuous future productivity. Since being innovative as such is, at least in many disciplines of the life sciences,^9^ nothing that researchers can capitalise on (see also Rushforth, Franssen, and de Rijcke 2019), it often only counts if it can be made durable in terms of publications or employability. As such, the first important argument of this paper is that it is indispensable to analyse demands for innovativeness distinct from those for competitiveness and productivity, and to play close attention to the partially conflicting pulls they create for researchers.

The second central argument I have made is that prevailing valuations of innovativeness, and especially the *de-valuation* of taking second steps and doing continuous work on one topic they entail, bring along considerable frictions with what researchers perceive as a valuable progress of knowledge and with the pursuit of more long-term epistemic agendas. In relation to this, I have argued that current demands for innovativeness seem to be hardly grounded in the complex realities of scientific practices but entail a rather short-sighted and simplified imagination of processes of knowledge production which differs fundamentally from more long-term and distributed understandings held by researchers.

This paper then provides a contribution to studies which aim to analyse the consequences of contemporary research governance in several ways. First, it highlights that it is important to not only investigate and challenge demands for researchers to be productive and competitive, as many previous studies have done (such as Burrows 2012; Fochler et al. 2016; Müller and de Rijcke 2017) but that demands for innovativeness should be investigated with the same critical scrutiny. Thereby, it also adds a more fine-grained perspective to studies which have suggested that innovative research is impeded as a consequence of neoliberal regimes of research governance (Fochler, Felt, and Müller 2016; Franssen et al. 2018; Müller and de Rijcke 2017). While my analysis shows that this holds true to some extent, since researchers constantly need to balance being innovative and risky with requirements for remaining productive and hence competitive, I have sketched a somewhat opposite, yet equally problematic dynamic—where researchers are demanded to constantly move on to innovative topics, which hinders the continuous in-depth exploration of certain problems. This resonates with what has been described by Whitley et al. (2018), namely that a lack of “protected space” for research is likely to impede actual scientific innovations in the long-term, in the sense of contributions that affect many researchers from different fields. As such, my analysis has important implications for research governance. It highlights that while innovation incentives are certainly important, it is equally relevant to guarantee “protected spaces” for research to progressively build a knowledge base on certain issues and to appreciate such continuous work in different contexts in which research is evaluated. As long as funding programmes adopt rather short-sighted notions of innovativeness, coupled with demands for immediate productivity, this is unlikely to produce the desired outcomes. Furthermore, also in the evaluation of researchers’ career trajectories as well as of publications it may be fruitful to re-consider valuations of innovativeness compared to the value that is ascribed to the continuous and in-depth pursuit of certain research topics.

Second, my analysis adds important insights to studies which have sought to investigate how research governance reshapes epistemic practices (Gläser and Laudel 2016; Kaltenbrunner 2020; Laudel 2006; Müller and de Rijcke 2017), beyond placeholders such as the impediment of innovative research. As I have shown, prevailing pushes for innovativeness can prevent researchers from conducting follow-up or validation studies, which can, for example, result in untested hypotheses floating around in the scientific landscape. I have illustrated that researchers have certain agency in shaping these epistemic developments and that they may find creative workarounds to align conflicting regimes of valuation. Yet, such possibilities are not always given, and some research endeavours may remain systematically unexplored within these dynamics. This can, to use the words of my interlocutors, result in the construction of “empty frames” instead of detailed “walls of knowledge”, and thus potentially in the production of certain kinds of selective ignorance (see e.g. Elliot 2013; Frickel 2014; Kourany and Carrier 2020). Importantly, my sample allows to demonstrate that these dynamics are perceived as highly problematic by researchers in both more basic and more applied fields of the life sciences.

Third, this paper makes a more conceptual contribution to the field of valuation studies, particularly with regards to research that studies (e)valuation practices in academia. Previous studies in this area have investigated how research produces different kinds of value (Dussauge, Helgesson, and Lee 2015; Pinel 2020), how research itself is evaluated and how these evaluation practices have constitutive effects (de Rijcke et al. 2016; Rushforth and de Rijcke 2015), and how researchers align, order, or displace different values in their work (Fochler, Felt, and Müller 2016; Helgesson, Lee, and Lindén 2016; Rushforth, Franssen, and de Rijcke 2019). However, hardly any attention seems to have been devoted to how certain kinds of research are being structurally *de-valued*—such as those taking second steps instead of adventurously moving into novel areas. As such, I suggest that in order to gain a more comprehensive understanding of the performative effects of (e)valuation practices, it is fruitful to pay more attention to the systematic de-valuation which can go along with a dominance of certain regimes of valuation.

Last, my research contributes to previous analyses which have highlighted that capitalist cultural logics are, also detached from actual economic relations, becoming increasingly embedded in the academic sphere (Fochler 2016; Kleinman 2010; Mirowski 2011). Through tracing the project order of worth, which Boltanski and Chiapello have described as the prevailing spirit of contemporary neoliberal capitalism, in the academic landscape, I have highlighted that not only market logics and principles of accumulation and assetization, but also the “spirit” of innovativeness essentially shapes current cultures of knowledge production—legitimising but also constraining processes of accumulation in the academic sphere and thereby “alter[ing] from within the meaning of scientific activity” (Muniesa et al. 2017: 86). Overall, such an understanding of the broader cultural logics and valuations that are embedded in current regimes of research governance and the ways in which they reshape epistemic practices then seems indispensable for discussions about how science should be governed, and for working towards a transformation of contemporary research cultures.

## Data Availability

Due to the nature of the empirical work reported and the related research ethics arrangements, the data is not made publicly available. Please contact the author for any requests or inquiries.

## Footnotes

1 Quoted from the funding guidelines of a major Austrian funding agency.

2 The *spirit* of capitalism is for Boltanski and Chiapello a prevailing ideology that justifies capitalist accumulation processes but simultaneously constrains them.

3 Because the aim of this paper is the investigation of demands for innovativeness, I pay less attention to the role of networking which Boltanski and Chiapello have identified as important to the project order of worth. While the ability to build and extend networks is crucial in the contemporary academic sphere as well, an additional analysis of this aspect would have diminished the clarity of my argument in the limited scope of this paper.

4 Due to physical distancing measures for Covid-19 containment, two of the discussions were held online. To ensure comparability, the design of virtual and physical focus groups was kept as similar as possible, and it turned out that the online format did not have any negative impact on discussion dynamics.

5 While all of the participating groups locate (parts of) their work in the sphere of ‘basic research’, especially the group from the field of plant breeding has equally many ‘applied’ projects and regularly collaborates with actors outside of academia such as industry partners.

6 The present paper indicates how valuations of innovativeness play out in different evaluative contexts, but it primarily aims to describe the overall regime of valuation arising from the interplay of these valuations across the contexts of publishing, funding, and career advancement. A more systematic conceptualisation of how valuations of innovativeness play out differently in these evaluative settings may be a valuable focus for further inquiry.

7 While the project-innovation regime could of course be considered in relation to different regimes of valuation present in the sphere of research, I focus on those ones that were most prominent in the accounts of my interlocutors and that seemed to cause most tensions for them.

8 When using this expression, I do not refer to a process of building cumulative objective knowledge through discovering things out there in the world—rather, I adopt the perspectives of the researchers I worked with and what they consider as important with regards to gaining a better understanding of a certain issue.

9 Certainly, disciplinary differences in valuation are crucial to take into account (see e.g. Hessels et al. 2019), and these dynamics may thus vary, and require separate investigation, in other scientific fields.
